# Biochemical and Structural Characterisation of a Novel D-Lyxose Isomerase From the Hyperthermophilic Archaeon *Thermofilum* sp.

**DOI:** 10.3389/fbioe.2021.711487

**Published:** 2021-08-06

**Authors:** Simone Antonio De Rose, Tom Kuprat, Michail N. Isupov, Andreas Reinhardt, Peter Schönheit, Jennifer A. Littlechild

**Affiliations:** ^1^ The Henry Wellcome Building for Biocatalysis, Biosciences, College of Life and Environmental Sciences, University of Exeter, Exeter, United Kingdom; ^2^ Institut für Allgemeine Mikrobiologie, Christian-Albrechts-Universität Kiel, Kiel, Germany

**Keywords:** sugar isomerase, lyxose, thermostable, crystal structure, industrial applications

## Abstract

A novel D-lyxose isomerase has been identified within the genome of a hyperthermophilic archaeon belonging to the *Thermofilum* species. The enzyme has been cloned and over-expressed in *Escherichia coli* and biochemically characterised. This enzyme differs from other enzymes of this class in that it is highly specific for the substrate D-lyxose, showing less than 2% activity towards mannose and other substrates reported for lyxose isomerases. This is the most thermoactive and thermostable lyxose isomerase reported to date, showing activity above 95°C and retaining 60% of its activity after 60 min incubation at 80°C. This lyxose isomerase is stable in the presence of 50% (v/v) of solvents ethanol, methanol, acetonitrile and DMSO. The crystal structure of the enzyme has been resolved to 1.4–1.7 A. resolution in the ligand-free form and in complexes with both of the slowly reacting sugar substrates mannose and fructose. This thermophilic lyxose isomerase is stabilised by a disulfide bond between the two monomers of the dimeric enzyme and increased hydrophobicity at the dimer interface. These overall properties of high substrate specificity, thermostability and solvent tolerance make this lyxose isomerase enzyme a good candidate for potential industrial applications.

## Introduction

In the last 50 years, the incidence of chronic “lifestyle” diseases such as diabetes, obesity, hyperlipidemia, and hypertension has increased rapidly throughout the world. These diseases are generally caused by the over intake of high sugar and high-fat foods. As a result, functional sugars with unique physiological benefits which can be used in the production of calorie-free sweeteners and nutraceuticals have attracted significant public attention (Zhang et al., 2017). Rare sugars can also be important building blocks for new drugs (Kwon et al., 2010).

Chemical synthesis of rare sugars requires strict reaction conditions, complicated purification steps, produces chemical waste, and has production safety issues. The application of a biocatalytic route for sugar production is more sustainable however it requires the application of specific enzymes. Also the stability of the required enzymes can be a limitation for their commercial applications. Thermophilic enzymes that are encoded within the genomes of microorganisms that grow at high temperatures can be used to overcome these problems. Enzymes from thermophiles are generally stable to higher temperatures and can also survive exposure to organic solvents (Littlechild, 2017). These thermostable biocatalysts can also be used at ambient temperatures where they have a lower activity but remain active for a longer time that allows them to be recycled for repeated turnovers. This principle has been applied for other industrial enzymes such as the thermophilic epoxide hydrolases (Ferrandi et al., 2018).

**Figure:**
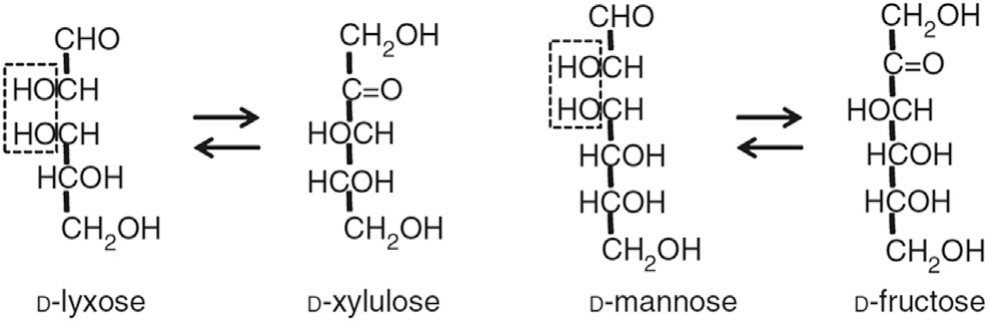


D-lyxose isomerase (LI, EC 5.3.1.15), is an important aldose-ketose isomerase reported to catalyze the reversible isomerization reaction between D-xylulose and D-lyxose, as well as between D-fructose and D-mannose (Scheme above) and several other D- and L-sugar substrates (Huang et al., 2018).

To date only a small number of LI enzymes have been characterized including those from *Aerobacter aerogenes* (Anderson and Allison, 1965), *Cohnella laevoribosii* (Cho et al., 2007a), *Serratia proteamaculans* (Park et al., 2010), *Escherichia coli* (van Staalduinen et al., 2010), *Providancie stuartii* (Kwon et al., 2010), *Bacillus licheniformis* (Patel et al., 2011), *Bacillus subtilis* (Marles-Wright and Lewis, 2011), *Dictyoglomus turgidum* (Choi et al., 2012) and *Thermosediminibacter oceani* (Yu et al., 2016).

The LIs play an important role in the microbial catabolism of D-lyxose and L-ribose which is linked to the pentose phosphate pathway (Cho et al., 2007b). Also it has been suggested that LIs have a role in the oxidative stress response (Marles-Wright and Lewis, 2011). D-lyxose is the precursor for the immune stimulant α-galactosylceramide and some anti-tumor agents, which can be used for treating some murine cancers (Morita et al., 1996; Takagi et al., 1996). Several LIs have been used for large-scale D-lyxose production from D-xylulose. These include enzymes from *P. stuartii* (Kwon et al., 2010), *S. proteamaculans* (Park et al., 2010) and *D. turgidum* (Choi et al., 2012).

Several LIs, including the enzyme from *Bacillus velezensis* (Guo et al., 2019), have also been applied in the production of L-ribose, a common intermediate of nucleoside based pharmaceuticals (Okano, 2009).

The LIs have been structurally classified as members of the cupin superfamily, which is one of the most functionally diverse classes of proteins. This superfamily includes isomerases and epimerases as well as non-enzymatic proteins which can be used in bacterial cell wall synthesis, as transcription factors and as seed storage proteins in plants (Dunwell et al., 2001; Dunwell et al., 2004). Two consensus amino acid sequences have been identified in the cupin family; a GX5HXHX3,4EX6G metal-binding motif 1 and a GX5PXGX2HX3N, motif 2, which are both found in the β-barrel fold that is characteristic of this enzyme family. These two motifs were defined before confirmation (Woo et al., 2000) of the previous prediction (Gane et al., 1998) that the two His residues and the Glu residue in motif 1, together with the His residue in motif 2, are involved in binding of the active site manganese ion.

Here we present the biochemical and physical characterisation of a thermostable lyxose isomerase from a *Thermofilum* species (TsLI) which shows high thermal stability and is highly specific for the substrate lyxose having little activity for other sugars. These are both properties that favour its use for industrial applications.

## Results

### Cloning, Expression, and Purification

To identify thermophilic LIs BLAST (Altschul et al., 1990) searches in genomes from hyperthermophilic archaea and bacteria, and metagenomes collected from thermophilic habitats, were performed using the sequences of biochemically and structurally characterized D-LIs as templates. This allowed the phylogenetic analyses of the new LIs to be carried out. The template sequences included LIs from the thermophilic bacteria *D. turgidum* (75°C) (Choi et al., 2012) and *C*. *laeviribosi* (70°C) (Cho et al., 2007a) and from the mesophilic bacteria *B. subtilis* and *E. coli*. A putative thermophilic LI was identified within the metagenomic sequences isolated from deep-sea hydrothermal vents and assigned to the *Thermofilum* species ex4484_79 (Locus tag: B6U94_07925). This protein showed high sequence identity to the LIs from *D. turgidum* (71%) and *B. subtilis* (59%) (Marles-Wright and Lewis, 2011) and lower sequence identity, 26%, to the *E. coli* LI (van Staalduinen et al., 2010).

**FIGURE 1:**
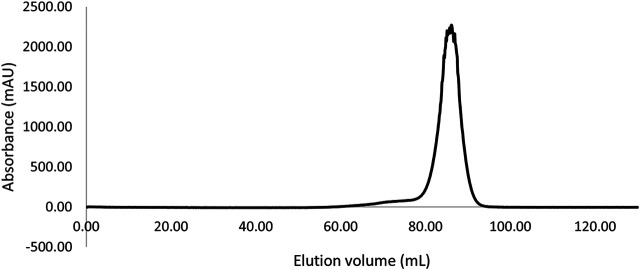
Size exclusion chromatography elution profile (Superdex 200 HiLoad 16/600) of the TsLI with an apparent molecular mass suggesting a dimeric state of the protein (∼46 kDa).

The gene coding for this putative LI was successfully cloned into the pET19b expression vector in frame with the N-terminal His 6x tag sequence under the control of the lactose inducible promoter. This allowed the LI protein to be successfully over-expressed in soluble form in *E. coli* Rosetta (DE3) pLysS. The enzyme was purified from the cell extracts by heat precipitation followed by Ni^2+^-NTA affinity chromatography with an excellent recovery yield (20 mg L^−1^). The affinity purified TsLI was confirmed to be a dimer of 2 × 23 kDa as eluted from the size exclusion chromatography column (Figure 1).

### Biochemical Characterisation

**FIGURE 2:**
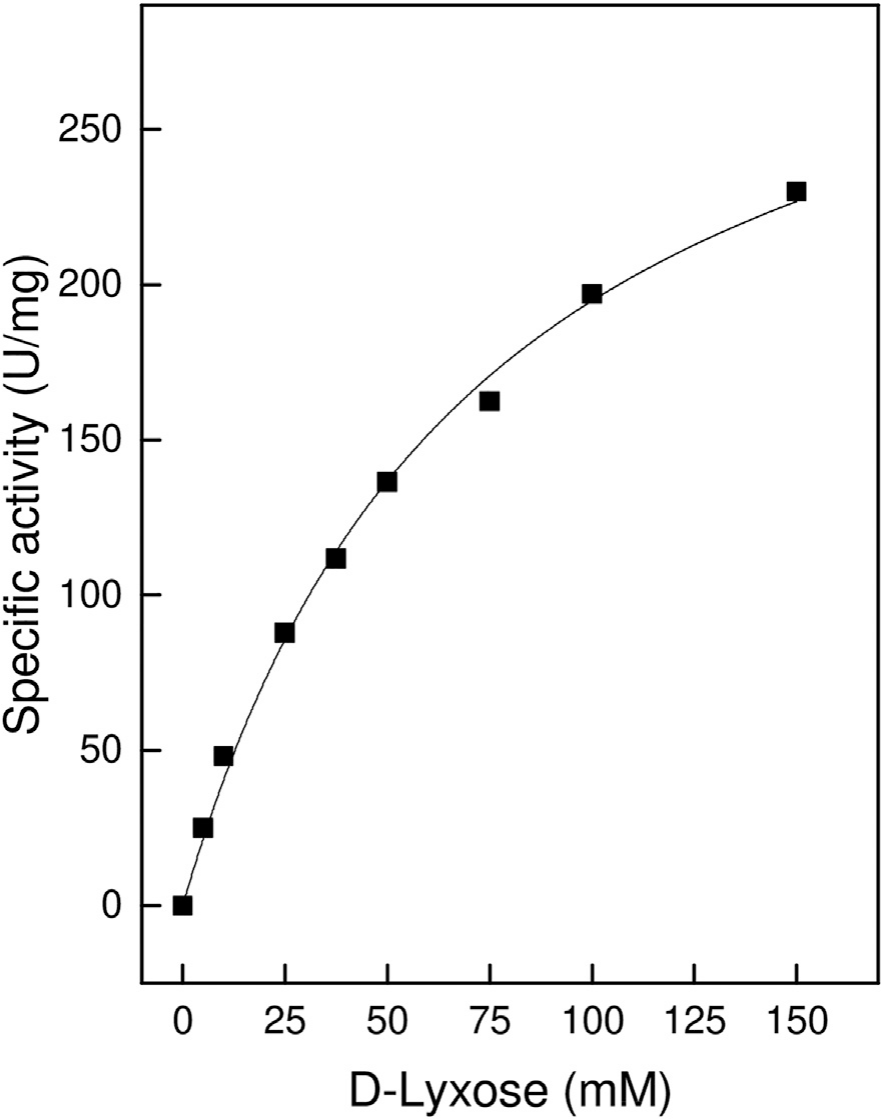
Specific activity ot TsLI towards the D-lyxose substrate, relative to the D-lyxose concentration. The assays (600 µL) were performed at 95°C in 50 mM BisTris buffer, pH 7.0, containing 1 mM MnCl_2_ and 6.7 µg enzyme. The kinetic constants were fitted to the Michaelis-Menten equation using the calculated standard errors; the Vmax and Km values were 338 ± 14.9 U/mg and 73 ± 6.6 mM, respectively.

When a range of D- and L-sugar substrates were assayed as potential substrates for TsLI it was shown that the enzyme catalysed, the conversion of D-lyxose to D-xylulose with a V_max_ of 338 U/mg and a K_m_ for D-lyxose of 74 mM at 95°C (Figure 2). In contrast to other characterised lyxose isomerases that have been reported to have a broader substrate specificity especially towards D-mannose (Huang et al., 2018), TsLI is highly specific for D-lyxose, with other aldoses (each used at 10 mM), including D-mannose, D-talose, D-xylose and L-ribose utilised at significantly lower rates (<2%).

**FIGURE 3:**
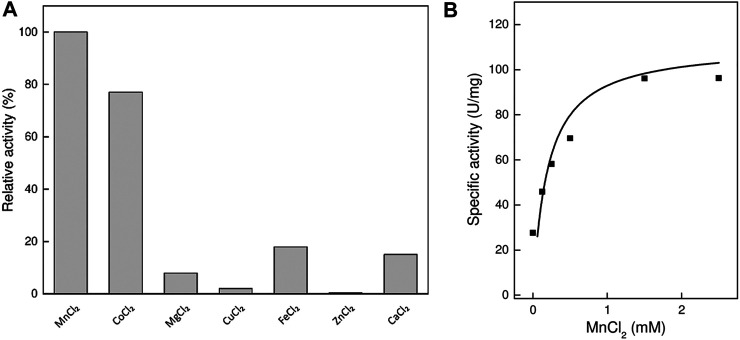
Effect of divalent cations on the activity of TsLI. **(A)** Relative activity with different divalent cations (1 mM) (100% = 60 U/mg) The assay (600 µL) was performed at 95°C in 50 mM BisTris buffer, pH 7.0, 50-mM D-lyxose and 6.48 µg enzyme. **(B)** Rate dependency on the MnCl_2_ concentration. The assays (400 µL) were performed at 95°C in 50 mM BisTris buffer, pH 7.0, 50 mM D-lyxose and 5.06 µg enzyme. The kinetic constants were fitted to the Michaelis-Menten equation using the calculated standard errors; the Vmax, and Km values were 105 ± 9.9 U/mg and 0.19 ± 0.08 mM, respectively.

Like most of the sugar isomerases, TsLI is strictly dependent on divalent cations, showing highest activity with Mn^2+^ ions with a K_m_ of 0.4 mM. The Mn^2+^ ions could be effectively replaced by Co^2+^ ions (79%). The enzyme showed less activity with other divalent metal ions, Fe^2+^ (20%), Ca^2+^ (13%) and Mg^2+^ (8%), and no activity (<1%) was detected in the presence of Cu^2+^ and Zn^2+^ ions (Figure 3).

### Thermoactivity and Thermostability

**FIGURE 4:**
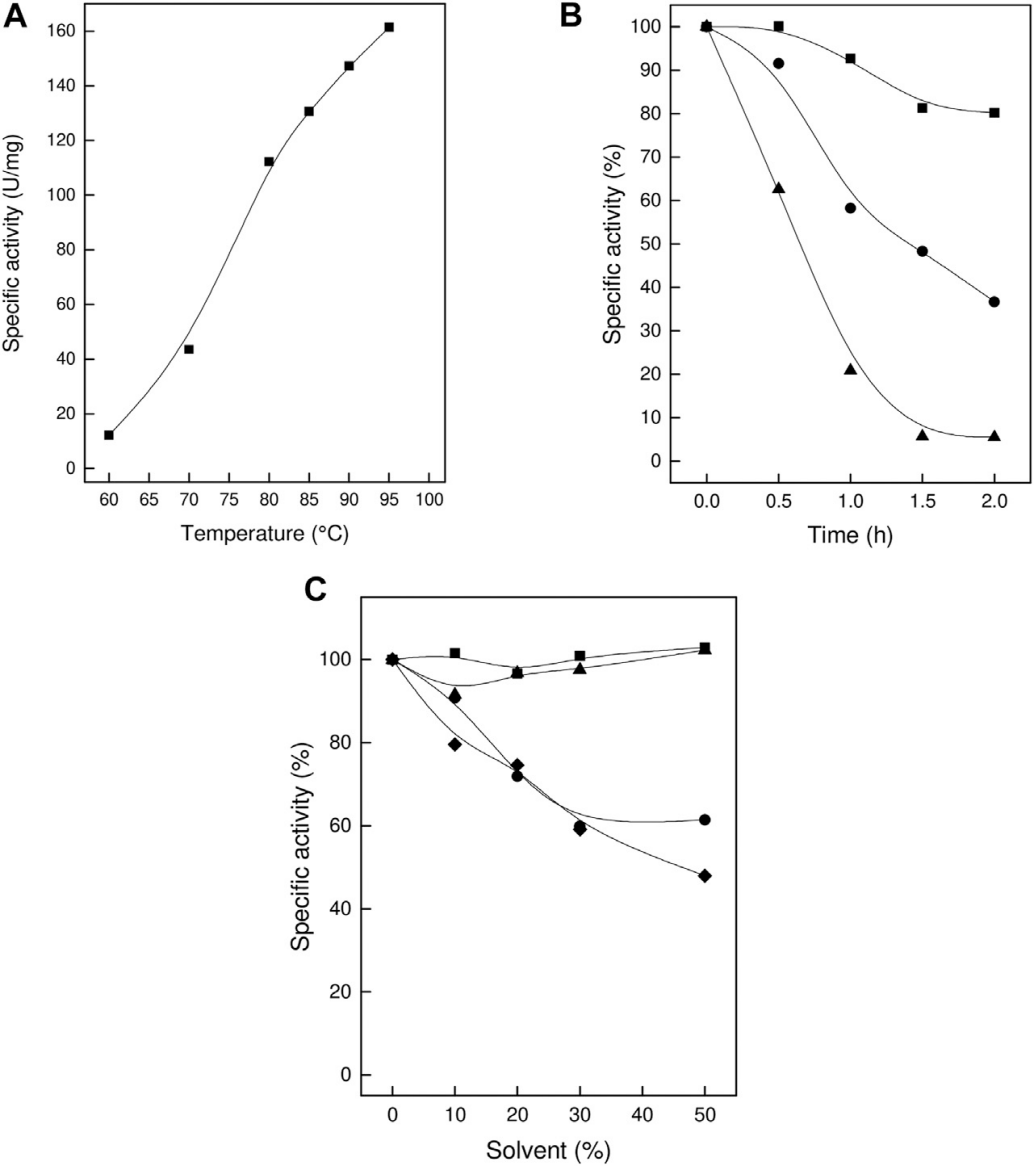
Thermoactivity, thermostability and solvent stability of TsLI. **(A)** Specific activity of D-lyxose isomerase measured between 60 and 95°C. **(B)** Residual activity of D-lyxose isomerase (in %) after preincubation for 2 h at 70°C (■), 80°C **(C)** and 90°C (▲) (100% 80 U/mg). The assays (600 µL) were performed in 50 mM Bis Tris buffer, pH 7.0, containing 1 mM MnCl_2_, 50 mM D-lyxose 6.7 µg enzymes. **(C)** Solvent stability. Residual activity (%) after 1 h incubation in the presence of the solvents (up to 50%) DMSO (■), ethanol **(C)**, methanol (▲) and acetonitrile (♦). (100% 110 U/mg). The assays (600 µL) were performed at 95°C in 50 mM BisTris buffer, pH 7.0, containing 1 mM MgCl_2_, 50 mM D-lyxose, and 6.7 µg enzyme.

The TsLI enzyme showed a temperature activity optimum of higher than 95°C (Figure 4A) and therefore constitutes the most thermoactive D-lyxose isomerase reported to date (Table 1). The thermostability of the enzyme was determined by analyzing the residual activity following pre-incubation of the enzyme for up to 2 h at different temperatures (Figure 4B).

**TABLE 1:** Comparison of the optimal temperature and of the metal dependencies of several characterised lyxose isomerases.

Organism	Temperature	Optimum metal	Reference
*C. laevoribosii*	70	Mn^2+^	Cho et al. (2007b)
*P. stuartii*	45	Mn^2+^	Kwon et al. (2010)
*S. proteamaculans*	40	Mn^2+^	Park et al. (2010)
*E. coli*	50	Mn^2+^	van Staalduinen et al. (2010)
*B. licheniformis*	40–45	Mn^2+^	Patel et al. (2011)
*D. turgiduma*	75	Co^2+^	Choi et al. (2012)
*T. oceani*	65	Mn^2+^	Yu et al. (2016)
*T. sp.*	>95	Mn^2+^	This study

After incubation for 2 h at 70°C, the enzyme did not show any significant loss of activity; after 1 h at 80°C and 90°C, the enzyme lost 40 and 80%, respectively, of its activity. The enzyme did not show any significant loss of activity upon storage at room temperature for three weeks.

### Solvent Stability

The organic solvent stability of TsLI was determined in the presence of ethanol, methanol, DMSO and acetonitrile. The enzyme was pre-incubated for 1 h at increasing concentrations (0–50%) of these solvents followed by determination of the residual activity. After incubation with 50% v/v of either DMSO or methanol, the enzyme retained full (100%) activity. After incubation in 50% v/v of either ethanol or acetonitrile, the enzyme showed a residual activity of about 50% (Figure 4C).

Together the data show that TsLI exhibits a very high stability towards temperature and organic solvents, which are important characteristics for potential applications of the enzyme for industrial biotechnology.

### Structural Characterisation of TsLI

The TsLI enzyme readily crystallized in several crystal forms in its ligand free form and in complex with the slowly reacting substrates mannose and fructose. The crystal structures of TsLI were solved by the molecular replacement (MR) method using the *B. subtilis* LI as a model. The structures reported here were isotropically refined to low R-factors with acceptable geometric parameters. The crystallographic data and model quality parameters are reported in Table 2.

**TABLE 2:** The TsLI data processing and structural refinement statistics.

LI	Native	D-fructose complex	D-mannose complex
Data collection statistics	—		
Beamline	I03 Diamond	I03 Diamond	I04 Diamond
Wavelength (Å)	0.9763	0.9763	0.9795
Space group	P2_1_	P2_1_	C222_1_
Unit cell parameters a, b, c (Å)	47.8, 86.9, 54.0	47.0, 86.7, 53.8	47.9, 121.0, 70.5
α, β, γ (°)	90.0, 112.9, 90.0	90.0, 112.3, 90.0	90.0, 90.0, 90.0
Resolution range (Å)^a^	39.25–1.67 (1.70–1.67)	86.65–1.35 (1.37–1.35)	37.66–1.58 (1.61–1.58)
Total reflections^a^	269,719 (4,520)	1,185,879 (57,654)	149,537 (7,011)
Unique reflections^a^	43,436 (1,266)	88,231 (4,299)	28,553 (1,411)
Completeness (%)^a^	92.2 (53.5)	100.0 (100.0)	99.9 (99.7)
Multiplicity^a^	6.2 (3.6)	13.4 (13.4)	5.2 (5.0)
R_meas_ (%)^a^ ^,^ ^b^	3.9 (179.5)	6.2 (608.2)	14.2 (249.5)
<I>/<σ(I)>^a^	19.4 (0.7)	14.9 (0.5)	5.6 (0.3)
CC_1/2_ ^a^ ^,^ ^c^	0.999 (0.326)	0.999 (0.333)	0.997 (0.311)
Wilson B-factor^d^ (Å^2^)	42.7	31.5	28.5
Refinement statistics	—		
R_work_	0.179	0.185	0.181
R_free_	0.211	0.213	0.223
No. of protein monomers in a.u.	2	2	1
Number of atoms	—	—	—
Macromolecules	2,981	3,144	1,594
Ligands/Metal ions	2	26	13
Solvent	293	353	244
Number of protein residues	354	365	177
RMS bond lengths (Å)	0.009	0.010	0.007
RMS bond angles (°)	1.61	1.84	1.54
Ramachandran favored (%)^e^	98.3	97.5	98.9
Ramachandran outliers (%)^e^	0.0	0.0	0.0
Clashscore^e^	3.3	4.0	4.9
Average B-factor protein (Å^2^)	40.1	34.0	23.3
Average B-factor ligands (Å^2^)	35.4	36.5	32.7
Average B-factor solvent (Å^2^)	49.3	43.5	36.3
RCBS PDB code	7NZO	7NZP	7NZQ

a Values for the highest resolution shell are given in parentheses.

b *R*_*meas*_**=** Σ_h_ [m/(m—1)]^1/2^ Σ_i_|*I*_*h,i*_—<*I*_*h*_>|/Σ_*h*_ Σ_i_ *I*_*h,i*_.

c CC1/2 is defined in (Karplus and Diederichs, 2012).

d Wilson B-factor was estimated by SFCHECK (Vaguine et al., 1999).

e The Ramachandran statistics and clashscore statistics were calculated using MOLPROBITY (Chen et al., 2010).

The structures of the native TsLI and the D-fructose and D-mannose complexes have been determined and refined to high resolution of 1.7, 1.4 and 1.6 Å, respectively.

**FIGURE 5:**
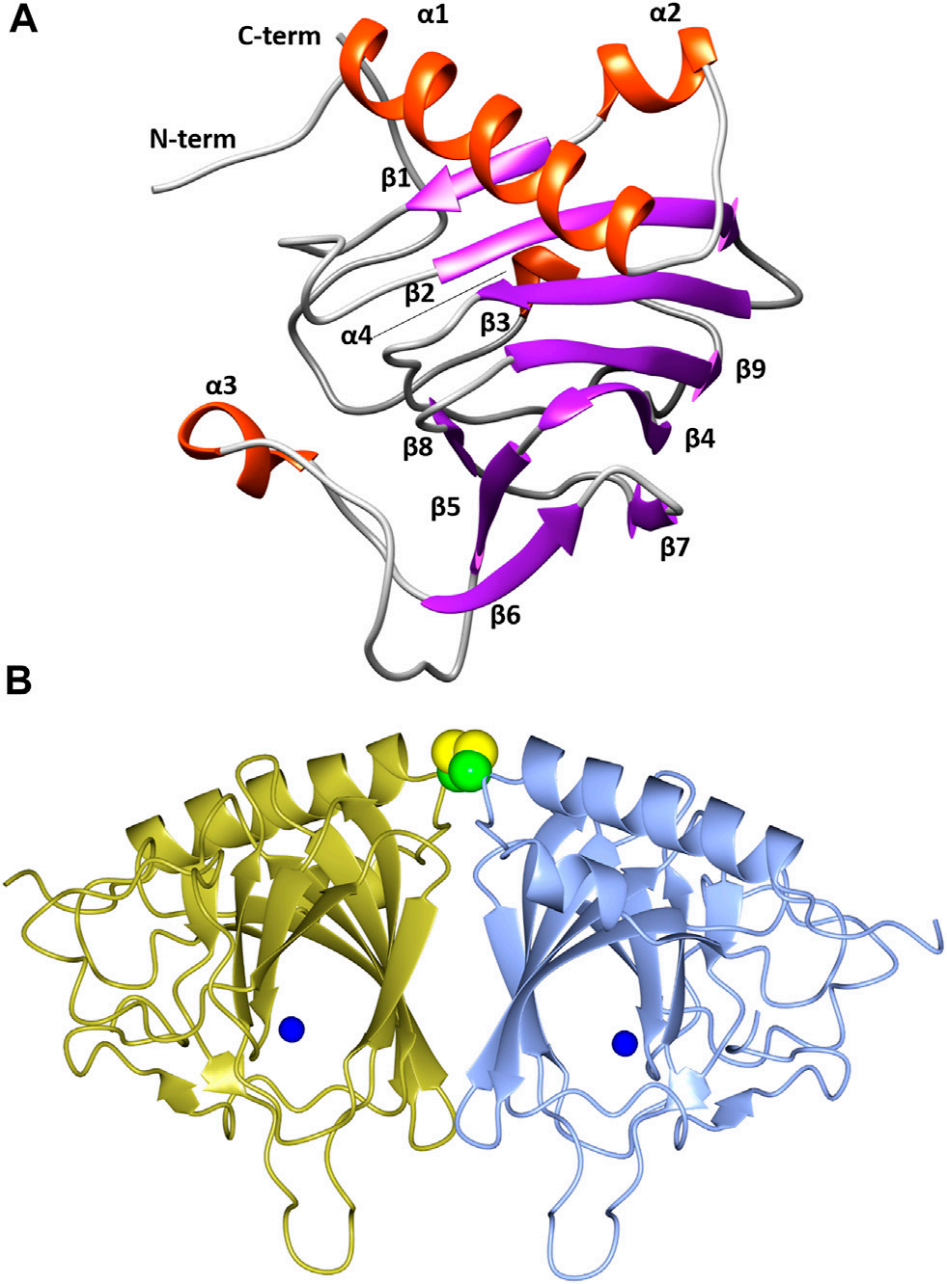
**(A)** A cartoon representation of the TsLI monomer with the secondary structure elements labelled. Figure was prepared with UCSF Chimera (Pettersen et al., 2004). **(B)** A cartoon representation of the cupin fold architecture of the TsLI dimer. The bound manganese ions are shown as blue spheres and the disulfide bond between the cysteines C22 of each monomer are shown as a space filling model. Figures 5B, 8–12 were prepared with CCP4mg (McNicholas et al., 2011).

The overall fold of TsLI is a cupin-type β -barrel (Figures 5A,B), with two α helices at the N-terminus, followed by the cupin barrel made up of two antiparallel β-sheets. There was strong electron density observed at the conserved cupin metal binding site, which was identified as manganese by the metal coordination and metal ligand distances. The metal identity was later confirmed using a X-ray fluorescence scan at beamline IO3 of the Diamond Light Source.

The β-barrel forms a deep pocket that is predominantly hydrophobic. The conserved manganese binding site is located within this pocket and is co-ordinated by the side chains of residues H75, H77, H143, and E88.

**FIGURE 6:**
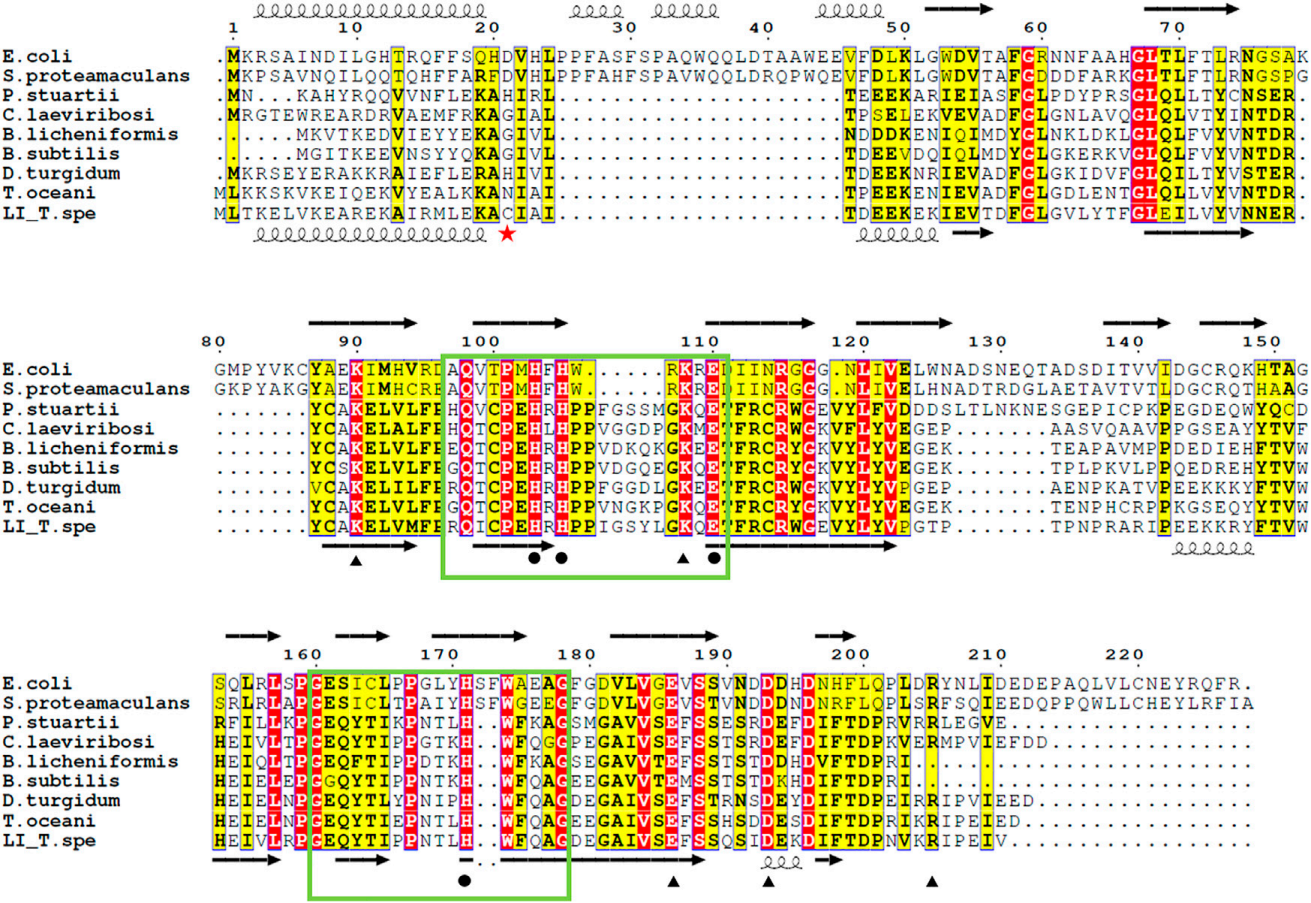
Alignment of the amino acid sequences of various LIs. Residues related to substrate binding sites (black circle), and those involved in both the metal coordination and substrate binding sites (black triangle), according to the ligand bound structures of *E. coli* LI bound to D-fructose (PDB 3KMH) and *B. subtilis* LI (PDB 2Y0O). Identical residues are in red while similar residues are in yellow, the green box highlights the cupin family motif 1 and 2. The red star indicates the cysteine 22 which is responsible of the disulfide bond connecting the dimer of the TsLI. The secondary structure of the D-LI from *E. coli* and TsLI are represented at the top and bottom of the alignment respectively with spring and arrow indicating α helices and β sheets. The alignment was carried out using ESPript 3.0 (http://espript.ibcp.fr/ESPript/ESPript/) (Robert and Gouet, 2014).

**FIGURE 7:**
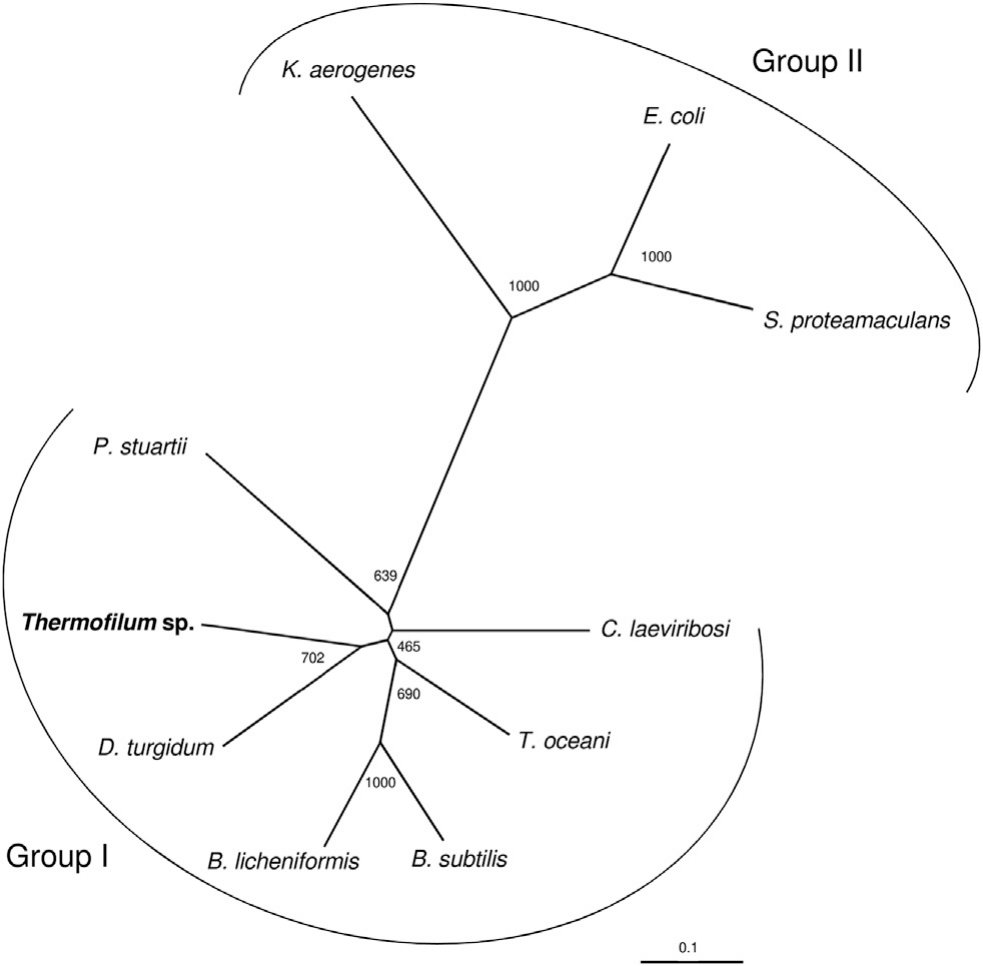
The phylogenetic relationship of TsLI with respect to other characterised bacterial lyxose isomerases from group I and group II type enzymes. The tree is based upon a multiple sequence alignment that was generated with ClustalO (Sievers et al., 2011). Numbers at the nodes are bootstrapping values according to neighbour joining. UniProt accession numbers are as follows: *C. laevoribosii* LI (GenBank accession no. ABI93960.1), *P. stuartii* LI (EDU58657), *B. licheniformis* LI (AAU22106.1), *B. subtilis* LI (AIY91703), *D. turgidum* LI (YP_002352606.1), and *T. oceani* LI (ADL08607.1) *S. proteamaculans* LI (BAJ07463.1), *Klebsiella aerogenes* LI (WP_154105566), and *E. coli* LI (Q8X5Q7).

As reviewed by Huang and colleagues (Huang et al., 2018) the LIs can be divided into two groups based on sequences, namely group 1 and group 2. Group 1 contains *C. laevoribosii* LI (GenBank accession no. ABI93960.1, 182 aa), *P. stuartii* LI (EDU58657, 183 aa), *B. licheniformis* LI (AAU22106.1, 167 aa), *B. subtilis* LI (AIY91703, 167 aa), *D. turgidum* LI (YP_002352606.1, 181 aa), and *T. oceani* LI (ADL08607.1, 181 aa). These enzymes have similar molecular weights, and they share 52–77% amino acid identity with each other. Group 2 contains the *S. proteamaculans* LI (BAJ07463.1, 228 aa), *Klebsiella aerogenes* LI (WP_154105566, 224 aa), and *E. coli* LI (Q8X5Q7, 227 aa). These enzymes have higher molecular weights than the LIs of group 1, and their amino acid identity is 71% over 79% coverage of the sequence. The TsLI belongs to group 1 according to the multiple amino acid sequence alignment shown in Figure 6 and in the phylogenetic tree (Figure 7).

**FIGURE 8:**
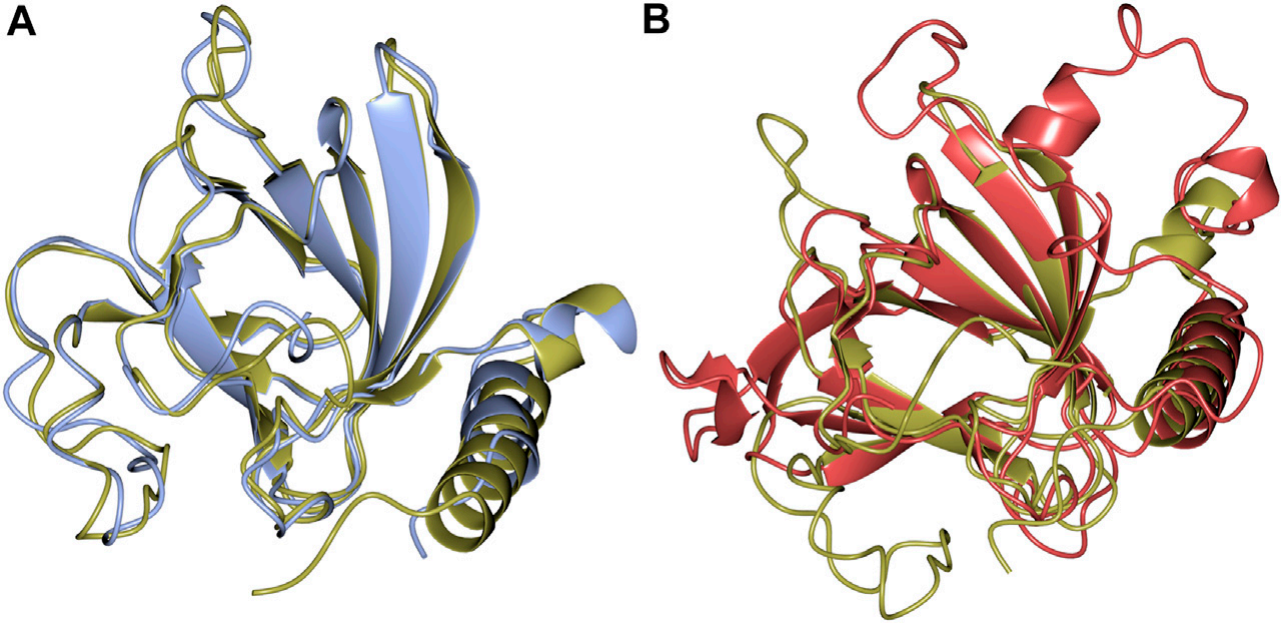
**(A)** Structural superimposition of TsLI (gold) and *B. subtilis* (PDB ID 2Y0O, cyan), which align with a RMSD of 1.3 Å over 169 aa out of 171 in total. **(B)** Structural superimposition of TsLI (gold) to *E. coli* LI (PDB ID 3KMH, red), which align with a RMSD of 1.7 Å over151 aa.

Structural alignments using the DALI server (Holm and Rosenström, 2010) revealed that there is high structural homology shared by TsLI and numerous cupin proteins. The most similar structures to TsLI in the PDB are the lyxose isomerase from *B. subtilis* and *E. coli*. The TsLI aligned well with the *B. subtilis* LI, with the two structures sharing 56% sequence identity and superposing with an RMSD of 1.3 Å (Figure 8A). The superposition of TsLI with the LI from *E. coli* (Figure 8B) revealed more differences as expected since these two enzymes belong to different LI groups. The TsLI has a more compact fold with shorter surface loops and has fewer secondary structure elements than the *E. coli* LI.

### The Active Site

**FIGURE 9:**
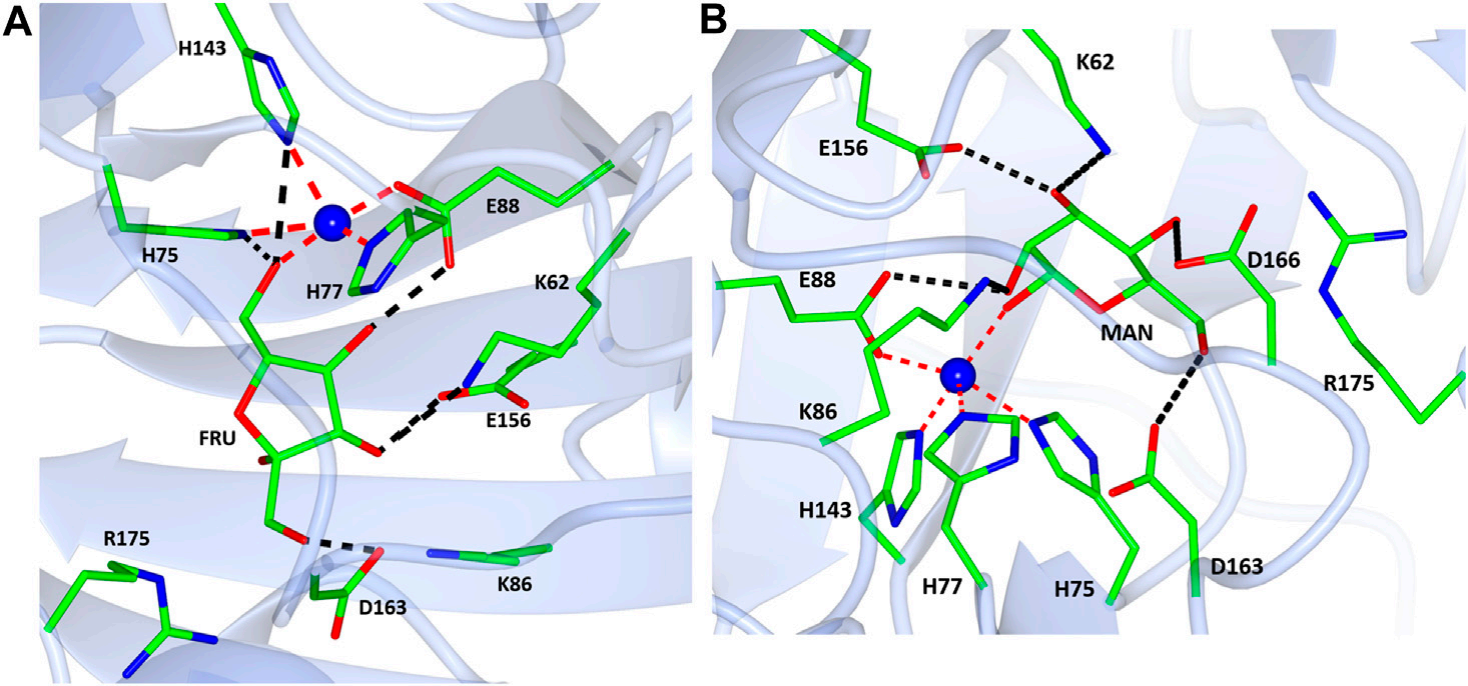
A diagram showing the active sites in the complex structures of TsLI with fructose **(A)** mannose **(B)**. The contacts of the ligands with the surrounding amino acid residues are shown as black dashed lines. The manganese ion is shown as a blue sphere with the coordination bonds shown in red.

**FIGURE 10:**
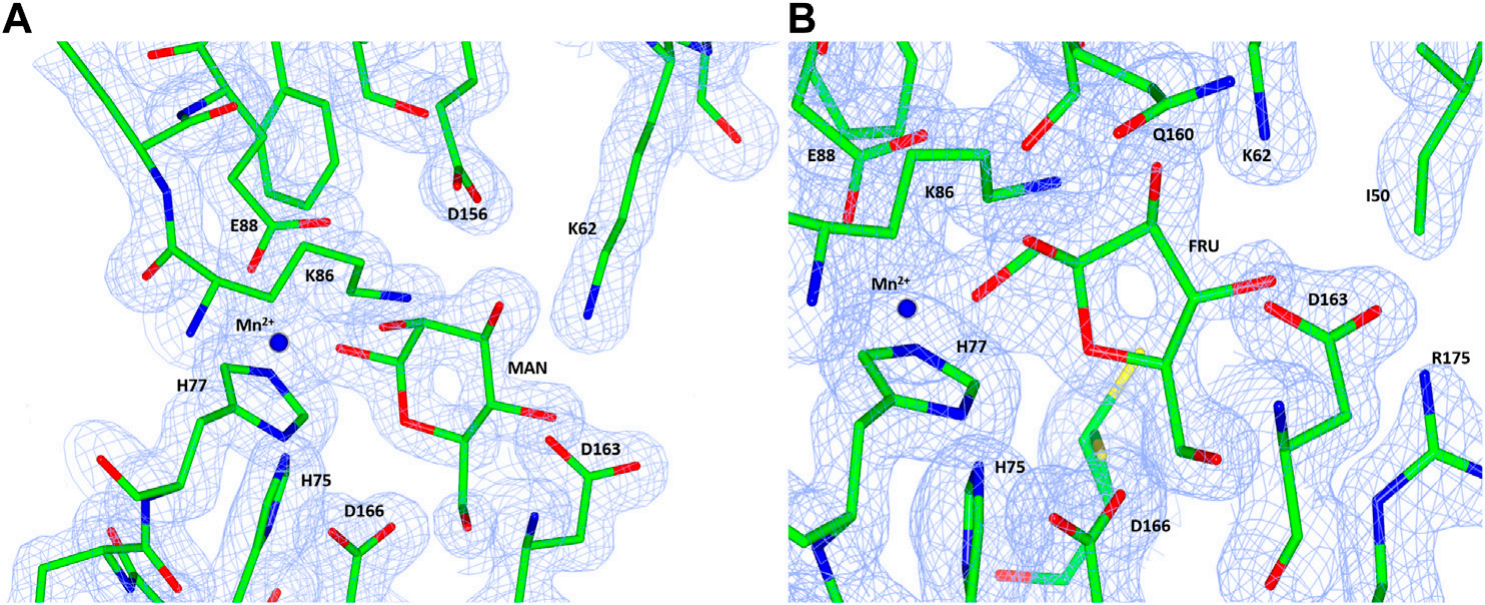
A diagram showing the electron density maps in the region of the mannose **(A)** and fructose **(B)** binding to TsLI. The 2Fo—Fc (blue) is contoured at 1.3 *σ*. The ligand and amino acid residues are shown as stick models and the manganese ion is shown as a blue sphere.

The native TsLI protein was crystallized without bound ligands and in complex with either 40 mM D-mannose or 15 mM D-fructose. Both cyclic sugar molecules are clearly defined in the electron density. They are bound in the active site through interactions with the amino acid residues K62, H75, H77, K86, E88, H143, E156 and D163 as well as the manganese ion (Figure 9). The two complex structures of TsLI with a bound β-D-mannopyranose or β-D-fructofuranose are very similar overall, and superimpose with an RMSD of 0.2 Å. It was possible to trap the slowly reacting sugar substrates, mannose and fructose in the TsLI active site due to their reduced turnover rates. The high specificity of the TsLI reaction towards the D-lyxose/D-xylulose pair seems to be due to the steric hindrance caused by the side chain of R175 that occupies part of the active site cavity (Figure 10). This contributes to a highly restrictive substrate binding pocket that is likely to reduce the binding of other pentose sugars and to also affect the positioning of the linear form of hexoses such as D-mannose for catalysis. The mesophilic *B. subtilis* LI has a shorter amino acid sequence at the C-terminus and does not have a residue at the equivalent position to R175, which opens up its active site. The equivalent R205 is present in the *E. coli* LI structure and is a part of a flexible loop which closes the active site pocket during catalysis, blocking solvent access (van Staalduinen et al., 2010), however R205 does not extend into the active site. The loop containing R175 in TsLI does not appear to change conformation between the non-ligated TsLI structure and the structures of TsLI sugar complexes. The quality of the electron density is illustrated in Figure 10. For the fructose TsLI complex the four potential tautomers were modelled into the electron density and the best fit was observed for the isomer β-D-fructofuranose. In the mannose complex the β-D-mannopyranose tautomer clearly fitted into the electron density.

Enzyme catalysed aldose–ketose isomerisation involves the transfer of a hydrogen between the C1 and C2 carbon atoms and it was proposed that this could happen by one of two mechanisms (Rose, 2006). As analysed in detail for phosphoglucose isomerases, including the metal dependent cupin type enzymes, hydrogen transfer proceeds either through a direct hydride shift (Swan et al., 2003) or by a cis-enediol intermediate (Berrisford et al., 2003) where the hydrogen is transferred in the form of a proton by a catalytic base. However, the hydride shift mechanism in cupin phosphoglucose isomerases is still a matter of debate with experiments using NMR and EPR adding evidence to support the cis-enediol intermediate mechanism (Berrisford et al., 2006) while quantum mechanics/molecular mechanics studies supporting the hydride shift (Wu et al., 2008). For the conventional metal independent phosphoglucose isomerases, it has been well established that these enzymes operate by the cis-enediol intermediate mechanism (Read et al., 2001; Swope et al., 2021).

**FIGURE 11:**
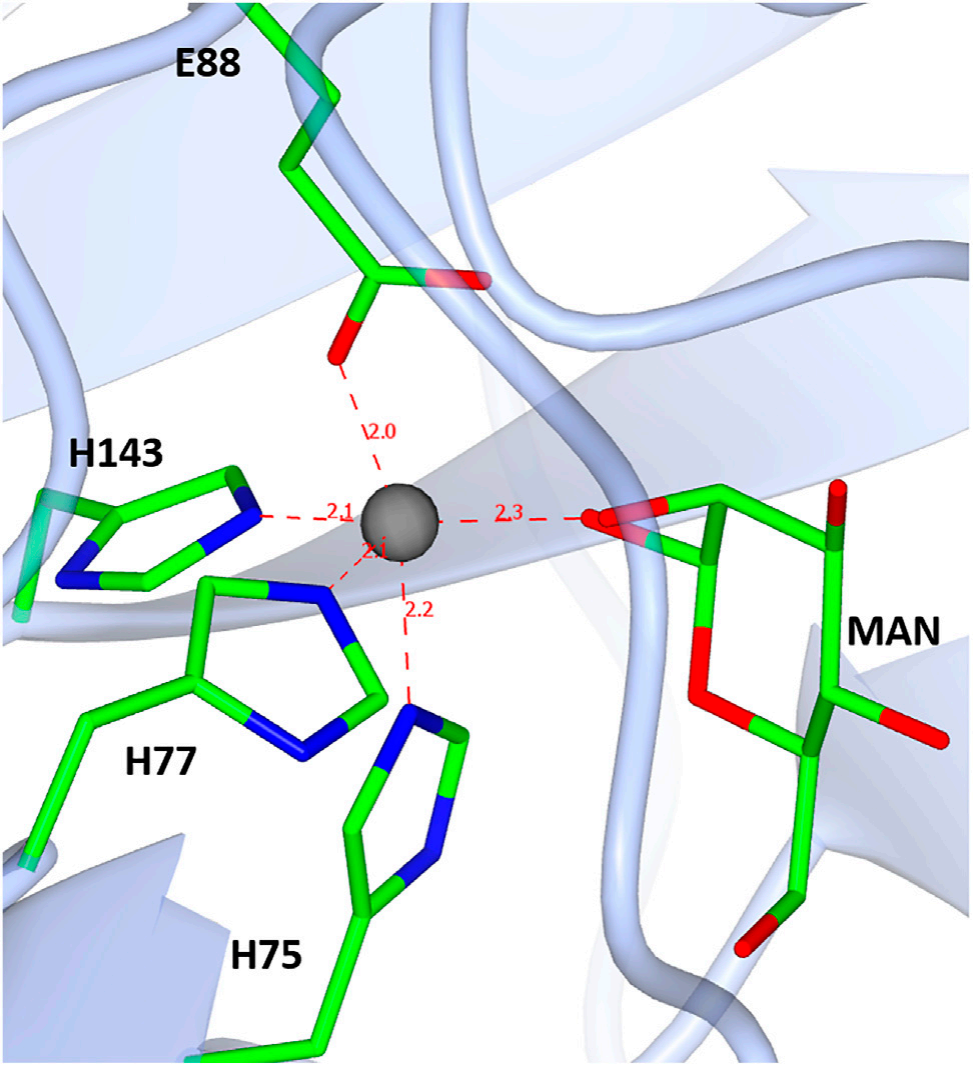
A diagram showing the D-mannose sugar bound in the active site of TsLI with the manganese ion (shown in red) co-ordinated to the three histidines H75, H77 and H143, one glutamic acid E88, and the manganese ion.

In the case of the TsLI and as already discussed by van Staalduinen and colleagues, the analysis of the active site residues indicate that the isomerisation is likely to proceed via a cis-enediol intermediate. The mechanism as proposed (van Staalduinen et al., 2010) starts with the substrate in its closed ring conformation. The metal coordinating histidine residue H75 could act as an acid catalyst for ring opening due to its vicinity to the O5 of the D-lyxose/D-xylulose (Figure 11). Once the ring is opened, another metal binding residue, E88, captures the proton, and a cis-enediol intermediate is formed. The subsequent intermediate stabilisation could be facilitated by the positive charged residue K86 or by the metal ion.

In their study van Staalduinen and colleagues mutated a key residue corresponding to E88 in TsLI which resulted in an enzyme with a significant decrease in activity. This residue was described as being fundamental for the metal binding and its mutation has been reported in several of the cupin enzymes to cause a severe decrease in activity (Dunwell et al., 2004; Hansen et al., 2005a; Hansen et al., 2005b). The mutation of the residues corresponding to K86, E156, and D163 completely prohibited the enzymatic activity either due to the disruption of the substrate stabilisation or the change of chemical characteristics of the active site pocket.

### Structural Basis for TsLI Thermostability

**FIGURE 12:**
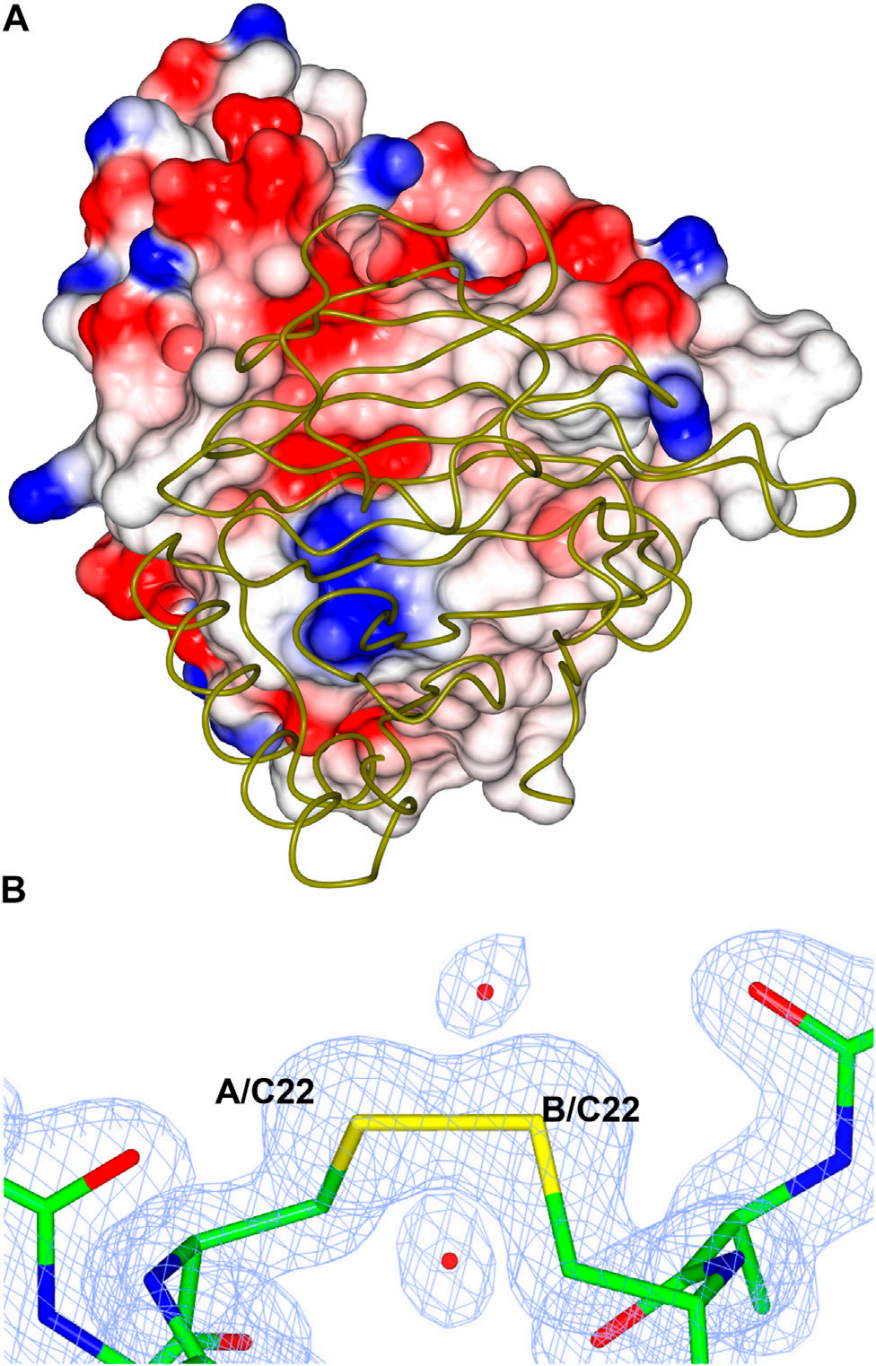
**(A)** A cartoon diagram showing the hydrophobic interactions at the dimer interface of TsLI. The electrostatic surface potential of one monomer of TsLI that has been rotated by 90° around the vertical axis from that presented in Figure 7 is shown together with the other monomer of the dimer shown as an α-C backbone (green). The areas of positive charge are shown in blue, with the areas of negative charge in red and the hydrophobic surfaces are represented in white. **(B)** Detail of the electron density showing the intersubunit disulfide bond formed by the cysteine 22 of each monomer. The 2Fo—Fc (blue) is contoured at 1.3 *σ* and the Fo—Fc map is contoured at 3.0 *σ* (green) and −3.0 *σ* (red).

This enzyme is the most thermoactive and thermostable lyxose isomerase reported to date, showing activity above 95°C and retaining up to 60% of its activity after 60 min incubation at 80°C. The TsLI dimer formation buries a surface area of 1,133 Å^2^, accounting for 13% of the total solvent accessible area of the monomer. The interface is stabilized by 16 hydrogen bonds and 8 salt bridges between the interacting monomers as estimated by PISA (Krissinel and Henrick, 2007). The dimer interface of TsLI is largely hydrophobic and contributes to the enzyme stability at elevated temperatures (Figure 12A). This feature is also seen for the dimer interface of the thermophilic transaminase from *Sulfolobus solfataricus* (Sayer et al., 2012) and the thermophilic epoxide hydrolase (Ferrandi et al., 2018). Most residues at the dimer interface are conserved between TsLI and *B. subtilis* LI, however in TsLI the respective F157 and A61 from the neighbouring subunit form extensive contacts, where the aromatic ring of F157 sits tightly on the side chain of A61. The contacts between the structurally equivalent M151 and S55 in the *B. subtilis* LI are limited and less hydrophobic. A general amino acid composition comparison between the two enzymes reveals that TsLI has a higher overall proportion of hydrophobic residues than *B. subtilis* LI (38.8% compared to 33.2%).

Furthermore, the two monomers of TsLI that form the active dimer are connected by a disulfide bond formed between the two cysteines at residue 22 of each subunit providing substantial thermostabilisation (Figure 12B). The disulfide bond is protected from the reducing environment within the cell by the nearby lysine residues at positions 20 in each monomer. This kind of protection has been previously observed in another hyper-thermophilic enzyme, carbonic anhydrase from a bacterial *Thermovibrio* species where two disulfides stabilize a tetrameric form of the enzyme (James et al., 2014). Disulfide bonds clearly do occur in intracellular proteins and are maintained despite the redox potential in the cell. The disulfide bond in TsLI is sufficiently buried to hinder the interaction with reducing agents, such as thioredoxin or gluthathione, allowing the disulfides to maintain their oxidized state. Previous studies in our group have found that the formation of intersubunit disulfides are used for stabilisation of pyrrolidone carboxyl peptidase enzyme from the thermophilic archaeon *Thermococcus litoralis* (Singleton et al., 1999). It has now become clear from crystallographic studies including that of the *Aeropyrum pernix* thermophilic alcohol dehydrogenase (Guy et al., 2003) and a variety of bioinformatic studies (Mallick et al., 2002) that intracellular protein disulfides are likely to exist in the aerobic archaea *Aeropyrum pernix* and *Pyrobaculum aerophilum*.

A disulfide bond in dimer formation of the *E. coli* LI between cysteines at positions 86 has also been reported. Since the TsLI and the *E. coli* LI enzymes belong to different groups of lyxose isomerase they cannot be directly compared since their molecular weight and sequence is very different (Figure 6). However, the related mesophilic group 1 *B. subtilis* LI, does not contain a disulfide bond at the dimer interface.

The TsLI is stable in the presence of 50% (v/v) of the organic solvents ethanol, methanol, acetonitrile and DMSO. These properties are consistent with the fact that if proteins are more thermostable they are also more solvent stable due to their more compact structure (Littlechild et al., 2007; Littlechild et al., 2013).

## Conclusion

A novel LI enzyme (TsLI) from the cupin family has been identified from metagenomic samples from deep-sea hydrothermal vents. This new thermostable enzyme has been cloned and over-expressed in *E. coli* and has been characterized both biochemically and structurally. The TsLI is the most thermoactive LI characterised to date with an optimal activity above 95°C, and it has the ability to retain 60% activity after 60 min incubation at 80°C. The TsLI has many of the molecular features that are known to contribute to thermostability including increased hydrophobic interactions, shorter surface loops, higher percentage of residues in α-helices, and a disulfide bond between the cysteine 22 of each monomer stabilizing the dimer. The TsLI shows an unusually high specificity for D-lyxose in comparison to D-mannose. This high specificity has enabled the slowly reacting substrates D-mannose and D-fructose to be trapped in the active site of TsLI in crystal complexes. The reduced activity of the enzyme with D-mannose is suggested to be due to the steric hindrance of R175 that makes the active site cavity too restrictive to bind other pentose sugars and restricts the hexose ring opening that is necessary for turnover. The TsLI is stable in the presence of 50% of the organic solvents, ethanol, methanol, acetonitrile and DMSO. These properties make TsLI a suitable candidate for new sustainable biocatalytic industrial applications.

## Materials and Methods

### Cloning and Over-expression of Recombinant D-Lyxose Isomerase From *Thermofilum* sp.

For heterologous expression of D-lyxose isomerase from *Thermofilum* species the pET system (Novagen, Madison, WI, United States) was used. The encoding gene was synthesized by Eurofins (Ebersberg, Germany) and cloned into pET19b. The resulting plasmid was multiplied in *E. coli* XL1-Blue MRF’ and used for transformation of *E. coli* Rosetta (DE3) pLysS. For expression of His-tagged protein, the transformed *E. coli* Rosetta (DE3) pLysS cells were grown in LB medium at 37°C up to an optical density at 600 nm of 0.6 and expression was induced by adding 1 mM isopropyl β-D-thiogalactopyranoside (IPTG). After 5 h of further growth at 37°C, the cells were harvested by centrifugation and stored at −20°C prior to enzyme purification.

The cell paste containing the TsLI was thawed and suspended in 50 mM Tris-HCl, pH 8.2, containing 300 mM NaCl and 5 mM imidazole and disrupted using a French press, followed by centrifugation. The cell-free extract was heat treated at 75°C for 30 min and heat-stable proteins were applied to a Ni-NTA column (Qiagen, Hilden, Germany) and eluted with 250 mM imidazole to obtain pure protein. The purified enzyme was then applied to a calibrated Superdex 200 pg HiLoad 16/600 size exclusion chromatography (SEC) column (Cytiva, Marlborough, MA, United States) and eluted with one column volume of 25 mM Tris-HCl pH 8.0, 150 mM NaCl at 1.0 ml min-1. The purity of D-lyxose isomerase and the molecular mass of subunits were analysed by SDS-PAGE. The protein concentration was determined by the method of Bradford (Bradford, 1976) with bovine serum albumin V as a standard.

### TsLI Activity Assay

The activity of D-lyxose isomerase was determined by a photometric assay measuring the formation of D-xylulose from D-lyxose using the cysteine carbazole test (Holzman et al., 1947; Dische and Borenfreund, 1951; Horecker, 1965). This discontinuous assay is suitable to analyze enzyme activities under extreme (hyper) thermophilic conditions (temperature optimum, thermostability) as well as enzyme stability in the presence of high concentrations of solutes. The kinetic constants of D-Lyxose isomerase, V_max_ and K_m_ values, were calculated with the Origin2015 software using the hyperbolic function according to the Michaelis-Menten equation.

### Crystallization

Prior to crystallization the TsLI was concentrated to ∼10 mg/ml using a 10 kDa membrane Vivaspin (Vivaproducts, Littleton, MA, United States) and microbatch crystallization trials were set up using an Oryx8 crystallization robot (Douglas Instruments, Hungerford, United Kingdom) using the JCSG+™ and PACT premier™ (Molecular Dimensions, Sheffield, United Kingdom) protein crystallization screens. For microbatch crystallization trials the droplet contained a 50:50 ratio of protein solution to screen and was covered with Al’s oil (50:50 mix of silicon and paraffin oils) before being stored at 20°C. Plates were set up in the presence of 40 mM of mannose or 15 mM fructose.

TsLI crystals appeared in microbatch plates within one week in several conditions. The crystals were harvested directly from the crystallization droplet and plunged into liquid nitrogen. The crystals used for the final structure refinement were harvested from the condition listed in Table 3.

**TABLE 3:** Crystallization condition of the different TsLI structures characterized in this study.

TsLI	Salt	Buffer	Precipitant	Ligand	Screen (Well)
Native	150 mM Potassium bromide	None	30% w/v PEG 2000 MME	None	JCSG+™ (G10)
Fructose bound	200 mM ammonium acetate	100 mM Bis-Tris pH 5.5	25% w/v PEG 3350	Fructose 15 mM	JCSG+™ (H10)
Mannose bound	None	100 mM SPG pH 6.0	25% w/v PEG1500	Mannose 40 mM	PACT premier™ (A3)

### Structure Solution

High resolution experimental data were collected from native monoclinic TsLI crystals on beamline I03 of Diamond Light Source Synchrotron (Didcot,United Kingdom) at 100 K in a stream of gaseous nitrogen using a Pilatus detector (Dectris) (Table 2). The data were processed by XDS (Kabsch, 2010) and DIALS (Waterman et al., 2016). The structure was solved using the molecular replacement pipeline MORDA (Lebedev and Vagin, 2015) with the best model identified as *B. subtilis* LI (PDB 2Y0O) which has a sequence identity of 58.5% when compared to TsLI. The model was refined using REFMAC5 (Murshudov et al., 2011) and rebuilt in COOT (Emsley et al., 2010). The fructose complex was refined in the same space group using data collected on I03 beamline. Ligand dictionaries for the four possible cyclic pyranose/furanose tautomers of D-fructose were prepared using JLIGAND (Lebedev et al., 2012) and the four tautomers were modelled into the electron density. The best fit to the electron density was observed for the isomer β-D-fructofuranose. The mannose complex crystallised in a base centered orthorhombic space group; data were collected on beamline I04 (Table 2). The refined native TsLI model was positioned by molecular replacement using MOLREP (Vagin and Teplyakov, 2010) in the mannose complex unit cell. The β-D-mannopyranose tautomer fitted the observed electron density. The PISA software (Krissinel and Henrick, 2007) was used for oligomeric state analysis of the TsLI models.

## Data Availability

The atomic coordinates and structure factors for the crystal structures of TsLI and its complexes have been deposited in the Protein Data Bank with the following codes: 7NZO for the native structure, 7NZP for the D-fructose bound structure, 7NZQ for the D-mannose bound structure.
